# The Medical and Physical Sciences

**Published:** 1853-04

**Authors:** W. P. Jones


### SELECTIONS.

From the Southern Journal of the Medical and Physical Sciences.

*The Medical and Physical Sciences.* By W. P. Jones, M. D.

It has been said, by one wiser than we, that "the Archetype of science is the Universe, and that it is in the disclosure of its successive parts that science advances from step to step." If this simple though comprehensive proposition be true, it becomes to every man, but especially the Physician, a matter of the greatest importance to observe these disclosures; and particularly in view of the short catalogue of medical men whose names have been rendered illustrious because of their devotion to the Physical Sciences.

We do not, and will not arrogate to ourself the importance of dictating to members of the medical profession, further than merely to intimate, (and this we do most respectfully,) that there is in no department of scientific or industrial pursuit, such an amount of varied and cultivated talent so unprofitably invested as in the learned profession of medicine.

Ordinarily, the *practice*, if not considered alone in a pecuniary point of view, does not pay. And where is the boasted literature of our profession?

Though the Physical Sciences have advanced with unprecedented rapidity within the last half century, it is very evident that we have not in medicine the classic literature foreshadowed fifty years ago. And it occurs to us as a somewhat remarkable fact, that notwithstanding we have inherited the learning of our fathers, we have been but reservoirs—not scientific dispensers. Inseparably connected as medical men are or ought to be, through their profession, with all the sciences, it becomes peculiarly their province to put forth a medical literature, worthy the name, worthy our predecessors, and the present age.

To do this most successfully, we should dissuade students from entering our offices, who have not acquired a thorough education; by which we will at least have learned whether they have the capacity and inclination to think—to think wisely—to think profoundly. If all would do this, in a few years we would have a literature of the highest order, commending itself to the judgment and cultivated admiration of the world.

Truth, immutable and unchangeable as the heavens, permeates all the sciences, and the separate departments of each are, equally with the whole, acted upon by a power far above the capacity of finite minds to comprehend. We, however, by searching may find out all, which in our present state concerns us to know; and whether we give utterance to our convictions or not, we can but feel within us that He who gave laws to Moses, on the summit of the mountain, now speaks to each of us, and not alone from the heavens, but from the summit of every mountain, and every table of stone composing the mountain, as well as from all other departments of the illimitable world. And no man, not even the boastful Atheist, can accomplish any useful purpose in life, unless he, too, studies the laws of nature and becomes a worker together with them.

We however know that few persons possess either the versatility of talent, the taste and inclination, or the physical and mental ability, to become eminently distinguished in every department of science; and consequently, to convince ourselves with reference to the importance of each of the great elements of learning, we must attend to the results of investigations among men of singleness of purpose. And to satisfy ourselves with regard to any one of the Medical or Physical Sciences, we have but to observe the conclusions arrived at by those who have devoted themselves exclusively to any one department, and see with what transcendent interest and importance they each invest their peculiar pursuit, and with what comparative indifference they regard all others.

While it is true that singleness of purpose characterizes those who obtain an intimate knowledge of the beauties, perfections and advantages of any science, it is also true, that nature has benevolently constituted other and more social natures, and probably less philosophic minds to search after and find the permeating tissues, the connecting links, which bind all the sciences, in one common harmonious brotherhood, and show that each is made to minister to the happiness of man, and all to the honor of the Divine Being.

We care not what may determine an inquiring mind to the investigation of any one of the sciences, it will be to him of all others the most interesting, the most improving, varied, and inexhaustible field for investigation and reflection.

Galen, the prince of Greek Physicians, though influenced by a dream of his father to turn his attention to the profession of medicine, upon that subject alone, is said to have written more than five hundred books.

Indeed, whatever in nature has for any considerable length of time engaged the philosophic mind, has in turn become the instrument of usefulness, the theme of admiration, and has effectually ministered to the sum of human happiness. Take for example the science of chemistry, how intensely interesting and almost infinitely diversified the great truths and principles presented there; and how vastly important their application to almost every department of business, if not to life itself. And who does not look to its future disclosures for the most important triumphs of Medicine?

In reviewing one's own bodily connections with elements of the past, the chemical and vital forces present themselves as peculiarly worthy the most minute consideration of the Medical Philosopher. A few years since and these bodies of ours had no existence. Elements which have become a part of our physical systems, differently combined. have been diffused throughout the world. Probably from every State in the Union—from China, West Indies, and Europe—from rill, river, and ocean—from plain, valley and mountain, these elements have come to us in organized substances, and after mastication, their nutritious products are further subjected to vital force or motion until they are introduced into the general circulation and become vital.

* * "How august,
How complicate, how wonderful is man!
How passing wonder, He who made him such!!
Who centred in our make such strange extremes,
From different natures marvellously mixed,
Connection exquisite of distant worlds."

It is also interesting and profitable, in a medical point of view, to contemplate our medical, physical, and intellectual relation to the geological character of the country in which we may chance more immediately to reside. As a science, separately considered, geology is worthy the profoundest investigation of the most herculean intellect, and as a department of American literature indispensable to the practitioner, because of its modifying, if not controlling influence over diseases peculiar to certain geological divisions.

In view of the general disclosures of geology, one may reasonably, or at any rate without being charged with lunacy, come to the conclusion that all nature was originally designed to become possessed of life, and those portions which have ever been subjected only to the laws of gravitation, but await their appointed time in the progressive transformation of matter to become vital. The annual round of spring, the action of the elements and natural affinities, bring about many new combinations. Chemical properties of stone and clay enter into the vegetable kingdom; the animal kingdom is dependent upon this, and through the medium of vital force and animal chemistry these properties of earth are transformed into living animal matter; and thus it is that the laws of nature are continually operating upon crude materials, refining and rendering them more easy of assimilation to man. The mineral kingdom furnishes nutriment to the vegetable; the vegetable to the animal kingdom, and vegetable and animal matter is the destined nourishment for man.

How vast, how interesting, how beautiful and sublime the economy of nature, which permits not even a drop of water to be lost, an autumn leaf to perish, or the smallest insect to decay, without subserving a valuable purpose!

"See dying vegetables life sustain;
See life, dissolving, vegetate again.
All forms that perish other forms supply;
By turns we catch the vital breath, and die."

The cultivation of the Medical and Physical Sciences as a system, though ever important to the life and happiness of men, has never yet met the reasonable demands of society. It is true, however, that at various times in the annals of medicine, such as Galen, Cullen, Cooper, Physic, Abernethy and others have by their scientific contributions adorned the profession of medicine. While Newton, Franklin, Humboldt and a host of others, have permeated the secret haunts and contributed largely to the elucidation of the Physical Sciences. Yet it is equally true, that the governing principles disclosed by the investigations of those men do not *seem* to sustain the mutual relations which the parts of a whole always sustain to each other. And why is this? Are there laws in nature without harmony? Principles without affinity? Are the elements by which we are environed so at war as to demand a mediator? We think not. They only require an adequate medium of intercommunication, and cultivated mind, which will ultimately subdue the world, must become that medium; and here a broad, beautiful and inviting vista opens up to the view of the medical inquirer. On either side, arrayed in rich and varied perspective, are the Medical and Physical Sciences, with here and there, a disintegrated though monumental pillar, the stand point for a time of some worthy predecessor in the healing art; but the accumulated mass of material on either hand remains wholly unavailable except as it may constitute a portion of one or another of the collateral sciences.

Who, in this age, can boldly enter the temple of the physical, and trace there the liniaments of the medical sciences? Who will reveal to us the intimate relation subsisting between the allied sciences? Or who, after observing the disclosures of nature, will attempt to show that the Trinity gave his similitude alone to man? and has now, elsewhere no counterpart in nature?

Chemistry, in some sense at least, is a spirit of truth alike common to the Medical and Physical Sciences, and takes hold of the principles and properties of both, and presents them to the mind of the medical philosopher. And it is thus that the scientific chemist, while elaborating philosophic truth for all classes of men, himself becomes one of the noblest ornaments of modern medicine.

Who, in fact, is now esteemed the most honorable man in the medical profession? Not the man in costly array who devotes himself to elegant leisure. Nor yet the man who pants for blood, and cuts where'er there's money. But, 'tis he who in his pupilage fitly studied prophylactic and therapeutic medicine, and whose consecrated energies are yet actively engaged in discovering the means of meliorating the condition of suffering humanity, and disclosing the hitherto hidden mysteries of medicine. 'Tis he who, while he sits, or rides, or reclines upon his couch, contemplates the relation which through his complicated physical organization he sustains to the earth upon which he walks, to the air he breathes, to the water he drinks and the food he eats, as well as to light, darkness, heat, cold, electricity, and the ten thousand other environments, which address themselves to his sentient nature.

In short, it is the man whose mind is richly imbued with the honor, the dignity, the responsibility and delicacy of his mission. Whose comprehensive capacities have been illuminated by the teachings of nature, and whose morals are guided by a purer revelation. All honor to such a man!

